# Milk and Milk-Contracts

**Published:** 1913-05-10

**Authors:** Everitt E. Norton

**Affiliations:** Medical Superintendent of Isleworth Infirmary.


### MILK AND MILK-CONTRACTS.

By EVERITT E. NORTON, M.D., D.P.H., etc., Medical Superintendent of Isleworth Infirmary.

### II.—The Source of Institution Milk Supplies.

The milk supplied under contract to institutions is brought, as is most of that retailed to the ordinary consumer in London, immense distances.

Apart from such supplies as now come from Ireland and from the nearer parts of the Continent, rail-borne milk is delivered in London from most of the English counties, from as far north as Cheshire and Lincolnshire, and as far west as Herefordshire and Devonshire; certain distant counties, such as Derbyshire and Wiltshire, furnishing large quantities. Dr. Thomas Orr found that milk is brought to Sheffield from places as far distant as 100, and to Hull from places as distant as 270 miles; and Dr. (now Sir) George Newman reported, in 1903, that 95 per cent. of the farms from which the supply of Finsbury was derived were situated at a greater distance than 100 miles from London.

It is obvious that the London morning milk delivery is (with the exception of a few special supplies) commonly of the previous afternoon's milking, which takes place some fifteen hours before the supply reaches the consumer. The afternoon delivery is, at best, from the morning milking, and the milk is some ten hours old; in the case of small retailers it is liable to be, wholly or partly, stored milk, older still. The milk has been procured under the well-known farm conditions.

Numerous scattered farms separately owned, practically without supervision of any kind, run by farmers often ignorant and concerned only in securing as full a supply as possible of milk of passable quality from a herd of cows whose one criterion of health is their milk-yielding capacity, the milk is only too often collected from unhealthy, improperly fed, dirty cows, in unsuitable surroundings, and under the dirtiest conditions.

Strained or unstrained, cooled or uncooled, the milk starts upon its journey in the time-honoured, unwieldy seventeen-gallon railway churn. No better argument in condemnation of this type of churn is needed than the fact that new churns, when now required, are almost always of different design. The old common churn (of which hundreds of specimens may be seen at any of our great railway stations) is worthy of a brief description. It is a tall conical vessel, the sides of which, at the point at which the loosely-fitting lid rests, slope somewhat outwards to form a rim, about three inches deep, rising above and surrounding the lid. To lift the full churn by the handles two men are required, and, as one man only is usually, during the various phases of its journey, available, it is "trundled" by him by means of this lip or rim, which constitutes the only and obvious hold for his dirty hands; the churns are also habitually used as seats by railway porters and others.

The dust, moreover, which falls upon the rim and upon the convex top of the lid settles down into the groove between them, and so falls into the churn or is shaken into it when the lid is removed. Should the churn be exposed in wet weather, rain trickles through the groove, carrying dust and dirt with it.

During the process of trundling and lifting the churn is necessarily somewhat tilted, and the milk may be seen to splash up around the lid, and to run in again with its acquired filth; if "ventilating" holes are provided, they afford an additional means of entry for dust. The milk is liable to be polluted by the dirty rim even if it be removed by a dipper, but especially if it be (as is customary) poured from the churn. The churns have, of course, no provision whatever for keeping the milk at any temperature other than that of the surrounding air. In carts, at the railway stations, and during the long journey the churns are exposed to dust. The railway vans are not only very seldom constructed, but are seldom used, for milk-traffic only, and the dust may be, according to the other uses to which they are put, more or less deleterious. The milk is usually long exposed to those conditions of shaking and high temperature, which are, in combination, eminently favourable to the rapid development of micro-organisms.

Strong objections have, with excellent reason, been made to the custom of pouring, handling, or measuring milk in such dirty and unsuitable surroundings as those of a railway station. It is to be hoped that such work is now (even in dealing with "accommodation" milk) seldom done at the London stations, and never in the case of supplies so large and so little varying in quantity as those sent to institutions. Similarly these supplies are, or should be, but little handled elsewhere before delivery. It is evident that the addition of chemical preservatives has steadily become less common, although quite recently serious attention has been called to the advertisement and use of a preparation, sold for dairy purposes, containing so dangerous a drug as sodium nitrite. On the other hand, milk is now taken with increasing frequency to "wholesale depots" and to the premises of middlemen.

It is not easy to ascertain precisely what is there done; there is no doubt that the milk is frequently mixed in bulk and the cream is reduced, by the removal of any excess, to a chosen standard by means of one of the separators which are now sold and used in such large numbers; this machine may act, at the same time, as a "centrifugal cleanser."

The pasteurisation of milk by the trade has been already referred to; whilst there has not arisen so definite a demand on the part of the public in this country for milk so treated as in America, milk intended for sale is often treated in some degree by heat in order to delay "turning," the degree of temperature and the time of exposure varying considerably. Any such treatment is less often adopted in the case of institution supplies than in that of milk intended for the retail trade, partly for the reason that some contracts plainly, and others indefinitely, forbid it, and partly because, the milk being more quickly delivered, it is less necessary to "preserve" it by such means.

Institution milk should, it appears, stand a better chance of direct delivery, within a comparatively reasonable space of time, in the churns in which it is placed at the farm, and should escape exposure to some contaminating influences.

